# Matrix Remodeling-Associated Protein 5 in Urinary Exosomes as a Potential Novel Marker of Obstructive Nephropathy in Children With Ureteropelvic Junction Obstruction

**DOI:** 10.3389/fped.2020.00504

**Published:** 2020-08-25

**Authors:** Qi Wang, Zhengzhou Shi, Xiaoyu Xing, Yiting Deng, Wenjie Li, Tianwei Xie, Dapeng Jiang

**Affiliations:** Department of Urology, Shanghai Children's Medical Center, Shanghai Jiao Tong University School of Medicine, Shanghai, China

**Keywords:** MXRA5, exosomes, biomarker, renal function, hydronephrosis

## Abstract

Recent investigations have described the use of urinary matrix remodeling-associated protein 5 (MXRA5) as a novel biomarker of kidney impairment in the setting of chronic kidney disease. In this study, we aimed to evaluate the possible clinical application of urinary MXRA5 as a useful non-invasive marker in the urine from the affected renal pelvis and bladder of children with ureteropelvic junction obstruction (UPJO). We conducted a prospective cohort study of patients aged <12 months with prenatally diagnosed unilateral UPJO who underwent dismembered pyeloplasty in 2018 or 2019, and a sex- and age-matched control group of healthy children. Blood urea nitrogen and creatinine levels were normal in all the patients. The whole urine and urinary exosomal concentrations of MXRA5 were measured by enzyme-linked immunosorbent assay. The correlations between bladder/renal pelvic MXRA5 levels and differential renal function (DRF) in the affected kidney were also determined. A total of 35 UPJO patients and 12 controls were enrolled in the study. There was no significant difference in whole-urine MXRA5 level between the controls and UPJO patients. However, the exosomal MXRA5 level was significantly lower in the controls than in patients with UPJO (*p* < 0.05). There were non-significant correlations between bladder and renal pelvis whole-urine MXRA5 levels and DRF (*R*^2^ = 0.1115, *p* = 0.05 and *R*^2^ = 0.3313, *p* = 0.0502, respectively). The strongest correlation was between exosomal MXRA5 level in the renal pelvis and DRF (*R*^2^ = 0.8128, *p* < 0.0001). Urinary exosomal MXRA5 level was significantly higher in children with UPJO than controls. Higher urinary exosomal MXRA5 levels were significantly correlated with lower DRF in the affected kidney in children with UPJO.

## Introduction

Congenital ureteropelvic junction obstruction (UPJO) is one of the most commonly encountered abnormalities that is responsible for persistent hydronephrosis in children. If left untreated, UPJO can lead to the progressive impairment of renal function (1). UPJO is a major cause of end-stage renal disease, which affects millions of children worldwide, highlighting the urgent need for early surgical intervention. However, the optimal timing and indications for surgical intervention in asymptomatic children with UPJO are controversial (2–6), given the possibility of spontaneous improvement in most hydronephrosis cases. Investigators have proposed intensive imaging protocols during the initial non-surgical management of patients, including serial ultrasound and diuretic renography (7, 8). The principal objective of surgical intervention is to reduce ipsilateral differential renal function (DRF), but it is costly, invasive, and exposes the child to ionizing radiation. Furthermore, nuclear scans often need to be repeated to assess the severity of damage caused by the obstruction (9). Therefore, there is an urgent need for the identification of a sensitive, specific, and non-invasive adjunct to such radiographic evaluation to assist decision making.

Multiple non-invasive urinary biomarkers have been evaluated for the identification of pathologic changes associated with UPJO (10–12). Several approaches, such as capillary electrophoresis and mass spectrometry, radioimmunoassay, and ELISA have been used to identify potential biomarkers (10, 13, 14). Previous studies have shown that the concentrations of urine biomarkers are associated with the severity of hydronephrosis and decrease following pyeloplasty (12, 15). Proteins can be secreted or shed into the urine from a number of cellular sources following UPJO-induced renal tissue damage, and may represent useful non-invasive biomarkers.

Urine proteins are a mixture of plasma-derived and kidney-derived proteins (16). They can be measured repeatedly and urine samples are readily obtained, but proteins of low abundance are difficult to quantify. Urine also contains various extracellular vesicles, of which exosomes are currently the most well-characterized. Urinary exosomes contain proteins and microRNAs that are produced by the kidney tissue and are increasingly recognized as a potential source of novel non-invasive biomarkers for renal diseases (17, 18), including for kidney injury induced by UPJO.

UPJO-induced renal injury is a complex process. Inflammation and fibrosis are key factors promoting the progression of obstructive nephropathy. Matrix remodeling-associated protein 5 (MXRA5) is a protein of unknown function belonging to the MXRA gene family that mediates kidney inflammation or fibrosis (19). The highest levels of MXRA5 have been detected in the kidney (20), and high tubular cell expression of MXRA5 was recently identified in human chronic kidney disease tissue (19). Moreover, MXRA5 is an anti-inflammatory and anti-fibrotic molecule that is up-regulated by transforming growth factor (TGF)-β1 in human proximal tubular cells (19). Given that proximal tubular dysfunction seems to develop relatively early in patients with UPJO, we hypothesized that MXRA5 might act as a potential diagnostic and prognostic marker for UPJO.

In this study, we aimed to evaluate urinary exosome MXRA5 levels in children with antenatally diagnosed unilateral UPJO, to analyze the relationship between the expression of urinary exosome MXRA5 and the deterioration of ipsilateral DRF induced by UPJO, and to compare the potential for whole-urine and urine-exosome levels of MXRA5 to be used biomarkers of obstructive nephropathy, using the example of UPJO.

## Materials and Methods

### Patients and Study Design

This prospective study was performed in accordance with the principles of the Declaration of Helsinki and was approved by the Shanghai Children's Medical Center Research Ethics Committee. Patients aged <12 months with prenatally diagnosed unilateral UPJO who had undergone dismembered pyeloplasty at the Department of Urology, Shanghai Children's Medical Center, Shanghai Jiao Tong University School of Medicine, in 2018 or 2019 were enrolled. Patients who were referred for repeat pyeloplasty were excluded from the study. Moreover, children who manifested maternal pathologies were also excluded from this study. Information regarding patient demographics, sonographic parameters, split renal function, serum urea and creatinine concentrations, intraoperative parameters, and surgical outcomes were collected. Most of the patients enrolled in the study had a prenatal history of hydronephrosis. Additionally, because differences in gestational maturity and the type of delivery between the groups might have influenced the findings, this information was also collected. The indications for pyeloplasty included increasing renal pelvic anteroposterior diameter (APD) and an obstructed renogram with DRF <40%.

We also enrolled sex- and age-matched normal controls with normal urinalysis, blood test, and renal ultrasound results. The exclusion criteria for both the UPJO and control groups were as follows: patients >1 year old; and patients with active or historical urinary tract infection, bilateral hydronephrosis, renal dysplasia, pelvic kidney, single kidney status, lower urinary tract abnormalities, or multiple anomalies. Written informed consent was obtained from the parents of all the patients and controls for the use of their children's urine for research purposes.

### Whole Urine and Urine-Exosome Preparation

**Figure 1:**
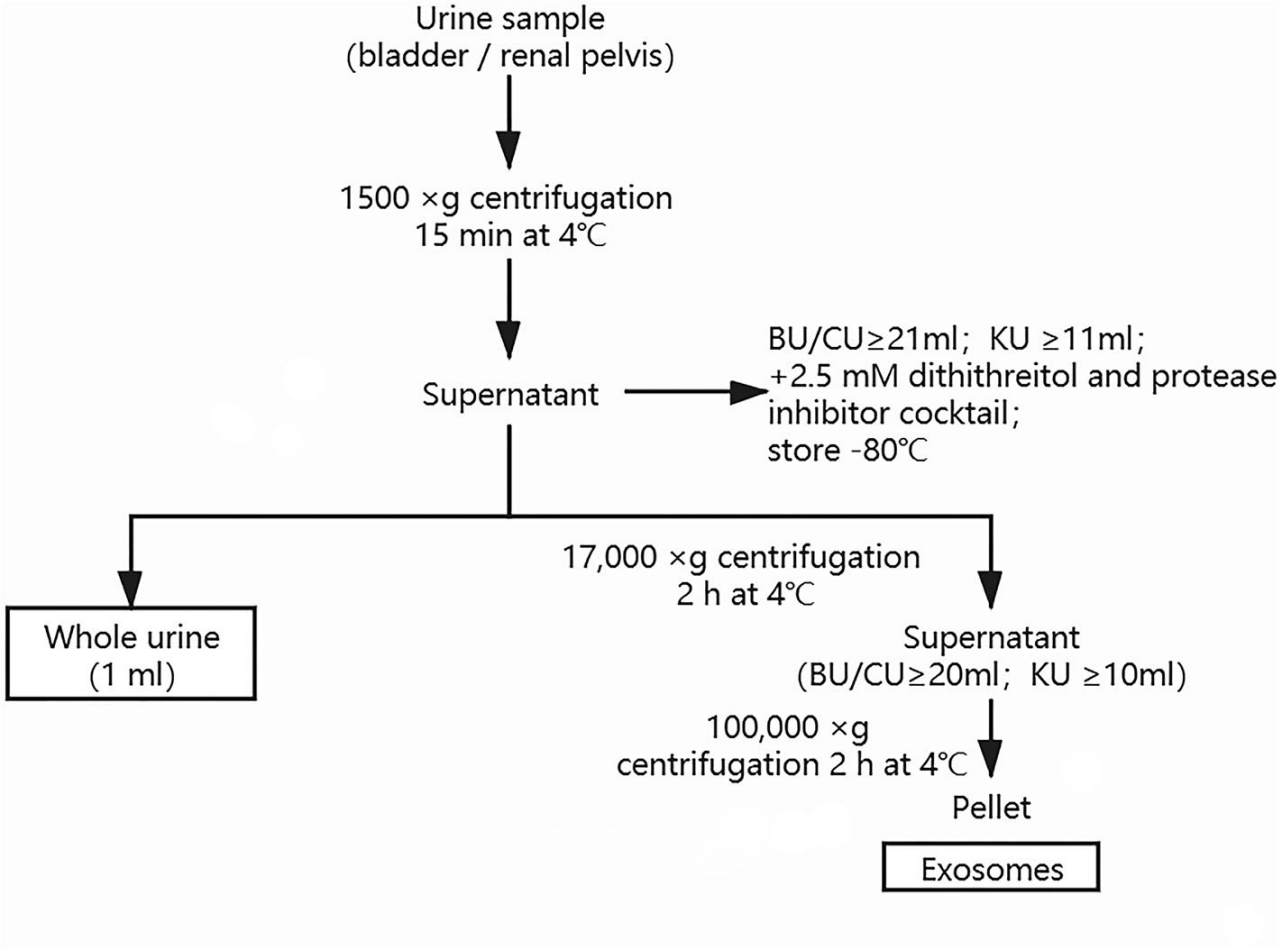
Methodology used for preparation of urinary exosomes.

Urine samples were obtained from the bladder by urethral catheterization before surgery in all the UPJO patients (sample BU). Voided urine samples were also obtained before surgery from these patients. Further samples were needle-aspirated from the renal pelvis during the surgical correction of the obstructed renal pelvis in some UPJO cases, before the relief of the obstruction (sample KU). Voided bladder urine samples were also obtained from the healthy children (sample CU). All the urine samples (≥21 ml for BU and CU; ≥11 ml for KU) were centrifuged at 1,500 × g for 15 min at 4°C to remove cell debris, then the supernatant was mixed with 2.5 mM dithiothreitol and a protease inhibitor cocktail and stored until use. An aliquot (1 ml) of the resulting supernatant was saved as a whole-urine sample and another aliquot (20 ml for BU and CU; 10 ml for KU) was used for exosome isolation by differential centrifugation and ultracentrifugation, as described previously (21). Urine samples (20 ml per patient or control for BU and CU; 10 ml per patient for KU) were vortexed thoroughly, then centrifuged at 1,500 × g for 10 min to remove cellular debris. The supernatants were then mixed thoroughly by vortexing and centrifuged at 17,000 × g for 15 min to remove cell organelles. The resulting supernatants were then ultracentrifuged at 100,000 × g for 2 h to obtain the urinary exosome pellet, which was resuspended in 200 μl of phosphate-buffered saline. The total protein concentrations of the urinary exosome preparations were quantified using a Micro Bicinchoninic Acid Protein Assay Kit (Pierce, US). The morphology and quality of the exosomes were evaluated by transmission electron microscopy and western blotting. We conducted nanoparticle tracking analysis (NTA) with a ZetaView PMX 110 (Particle Metrix, Meerbusch, Germany) and the corresponding software (ZetaView 8.04.02) to measure the exosome particle size and concentration. The exosome preparations were appropriately diluted using PBS to measure their concentrations and particle sizes, and the NTA measurements were made in 11 different positions. The ZetaView system was calibrated using 110-nm polystyrene particles. Whole-urine and urinary exosome samples were stored at −80°C for up to 3 months. The methodology used for preparation of the urinary exosomes is shown in Figure 1.

### Urinalysis

The MXRA5 level was measured using a commercially available ELISA kit (R&D Quantikine, Abingdon, UK). The MXRA5 levels in whole-urine samples were normalized to the urinary creatinine concentrations and are expressed as ng/mg creatinine. The urinary creatinine concentration was measured using an enzymatic method (Vitros 950, Johnson & Johnson, New Jersey,US). The MXRA5 levels in the exosomal fraction are expressed as ng/μg of exosomal protein. Each sample was analyzed in triplicate. The concentration of MXRA5 or creatinine in the urine is given as the mean value of triplicate measurements. To determine whether age and sex are associated with the level of MXRA5, the MXRA5 expression was compared in children of <3 and 3–12months, and between male and female children with UPJO and controls.

### Statistical Analyses

Data are shown as means ± standard deviations. Data were compared using analysis of variance (ANOVA), followed by Tukey's test. The relationships between variables were evaluated using Pearson's correlations. The prognostic value of exosomal MXRA5 in patients was analyzed by receiver operating characteristic (ROC) curves. Statistical analyses were carried out using SPSS 11.0 (Chicago, IL, USA). *P* < 0.05 was considered to represent statistical significance.

## Results

**Figure 2:**
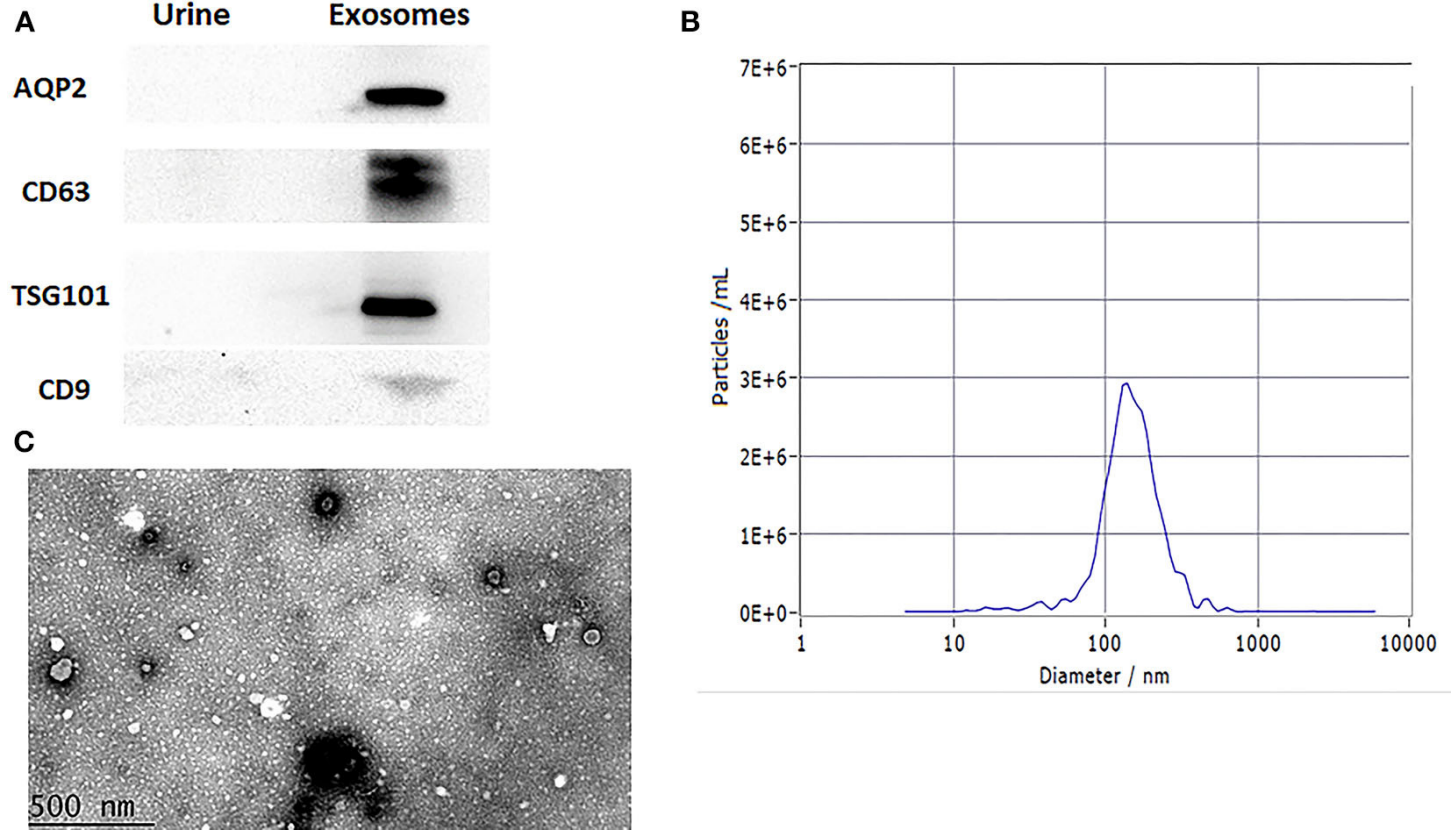
Characterization of urinary exosomes. **(A)** Western blotting showed the expression of AQP2, CD63, TSG101, and CD9 proteins in exosomes. **(B)** The size range of the particles. **(C)** Representative transmission electron micrographs, demonstrating the exosome morphology. Scale bar: 500 nm.

The levels of the surface markers AQP2, CD63, TSG101, and CD9 were measured in urine exosomes using western blotting (Figure 2A). NTA analysis accords with exosomal size as reported before (Figure 2B). Under transmission electron microscopy, the urine exosomes appeared as circular particles (Figure 2C).

The study included 35 patients with UPJO (30 male and 5 female children; median age 5.78 months, range 1–12 months) and 12 healthy controls (mean age 6.73 months, range 1–12 months). Among the patients with UPJO, the left kidney was affected in 25 children and the right kidney in 10. All the patients had been diagnosed with antenatal unilateral hydronephrosis. The demographics and clinical parameters of the UPJO patients are displayed in Table 1.

**Table 1:** Characteristics of the UPJO patients and controls.

**Variable**	**UPJO (*n =* 35)**	**Controls (*n =* 12)**	***P*-value**
Age at examination (months)	5.78 ± 4.8	6.73 ± 4.5	0.5511
Sex (boy/girl)	30/5	9/3	0.6839
Presence of albuminuria (n, %)	(1, 2.9%)	(0, 0%)	1.0000
Gestational age (term/preterm)	30/5	11/1	0.9744
**Type of delivery**			
(Natural vaginal delivery/ Cesarean delivery)	4/31	2/10	1.0000
Birth weight (g)	3,256 ± 411	3,321 ± 369	0.6305
Urea (mmol/L)	3.7 ± 2.9	4.5 ± 2.5	0.3988
**Creatinine (μmol/L)**			
<3 months	26.1 ± 13.7	28.2 ± 15	0.6567
>3 months	33.4 ± 15.2	39.7 ± 19.6	0.2565
Laterality (right/left)	10/25	-	
Kidney length (mm)	83.6 ± 9.7	59.6 ± 8.2	<0.0001
DRF (%)	41.4 ± 4.3	-	
APD (mm)	39.5 ± 7.6	-	
Parenchymal thickness (mm)	2.0 ± 0.6	-	
Calyceal dilatation (mm)	13.7 ± 4.95	-	

*Values are means ± standard deviations*.

Bladder urine samples were obtained from all the UPJO patients (BU, *n* = 35) and all the controls (CU, *n* = 12). Urine samples were also obtained from the renal pelvis of 12 of the UPJO patients (KU, *n* = 12). There were no significant differences in the urine levels of MXRA5 between children of <3 and 3–12 months of age, or between male and female children, in either the patient or control groups (Table 2).

**Table 2:** Expression of MXRA5 according to age and sex.

	**Age**			**Gender**		
**Parameter**	** <3 months**	**3–12 months**	***P*-value**	**Male**	**Female**	***P*-value**
**Whole urine MXRA5 (ng/mg creatinine)**						
CU	2.18 ± 0.24	2.29 ± 0.38	0.5831	2.014 ± 0.2	2.361 ± 0.36	0.0559
BU	3.19 ± 0.3	3.28 ± 0.45	0.4851	3.267 ± 0.36	2.98 ± 0.44	0.1184
**Exosomal MXRA5 (pg/μg exosomal protein)**						
CU	1.17 ± 0.35	1.4 ± 0.48	0.3854	2.39 ± 0.41	1.98 ± 0.285	0.1443
BU	2.74 ± 0.51	2.93 ± 0.37	0.2002	3.408 ± 0.55	3.02 ± 0.47	0.1471

*Values are means ± standard deviations*.

**Figure 3:**
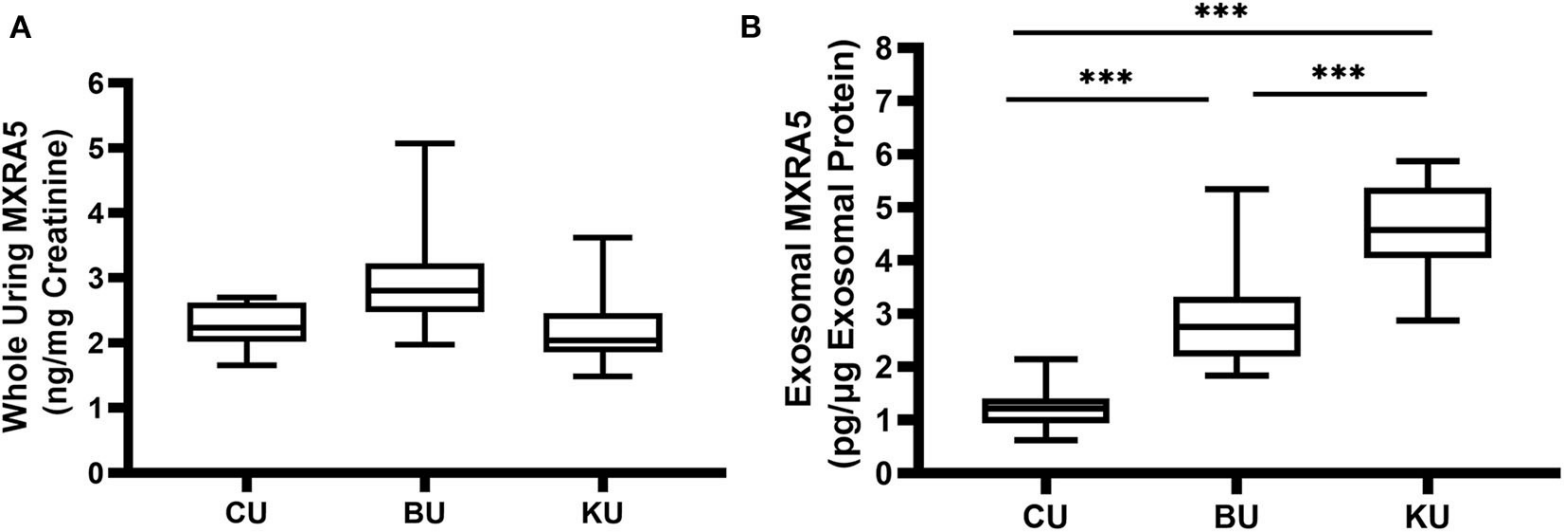
MXRA5 levels in BU, CU, and KU. **(A)** Whole-urine MXRA5 level. The whole-urine MXRA5 levels were similar in the controls and UPJO patients. **(B)** Urinary exosome level of MXRA5. The exosome levels of MXRA5 were significantly higher in the KU and BU than in the CU exosome samples. Data are presented as mean ± SD. ***P < 0.0001.

The whole-urine MXRA5 levels in the bladder tended to be lower in the controls (CU) than in the UPJO group (BU) (Figure 3A), but the difference was not significant. However, the exosomal MXRA5 levels in the bladder (CU) were significantly lower in the controls than in the UPJO group (BU) (*P* < 0.05) (Figure 3B).

We also obtained urine samples from the renal pelvis in the UPJO group (KU), but the median whole-urine MXRA5 levels in these samples were similar to those in the normal bladder samples (CU) (Figure 3A). However, the levels of MXRA5 were significantly higher (4-fold) in the KU than the CU exosome samples (Figure 3B).

**Figure 4:**
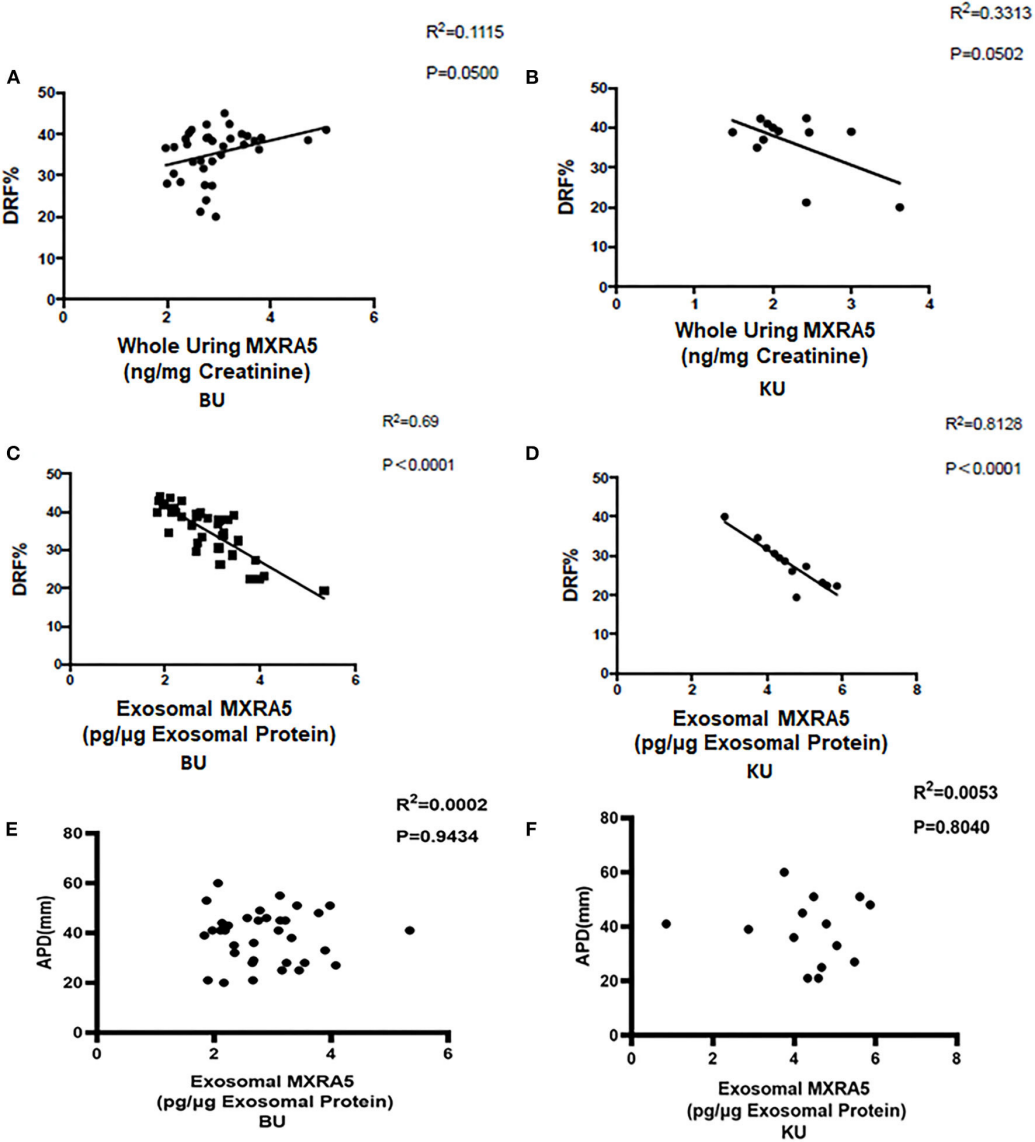
Pearson's correlations between urinary MXRA5 level and DRF in children in the UPJO group. **(A)** Correlation between whole-urine MXRA5 level and DRF (BU). **(B)** Correlation between whole-urine MXRA5 level and DRF (KU). **(C)** Correlation between the urinary exosome level of MXRA5 and DRF (BU). **(D)** Correlation between the urinary exosome level of MXRA5 and DRF (KU). **(E)** Correlation between the urinary exosome level of MXRA5 and APD (BU). **(F)** Correlation between the urinary exosome level of MXRA5 and APD (KU).

**Figure 5:**
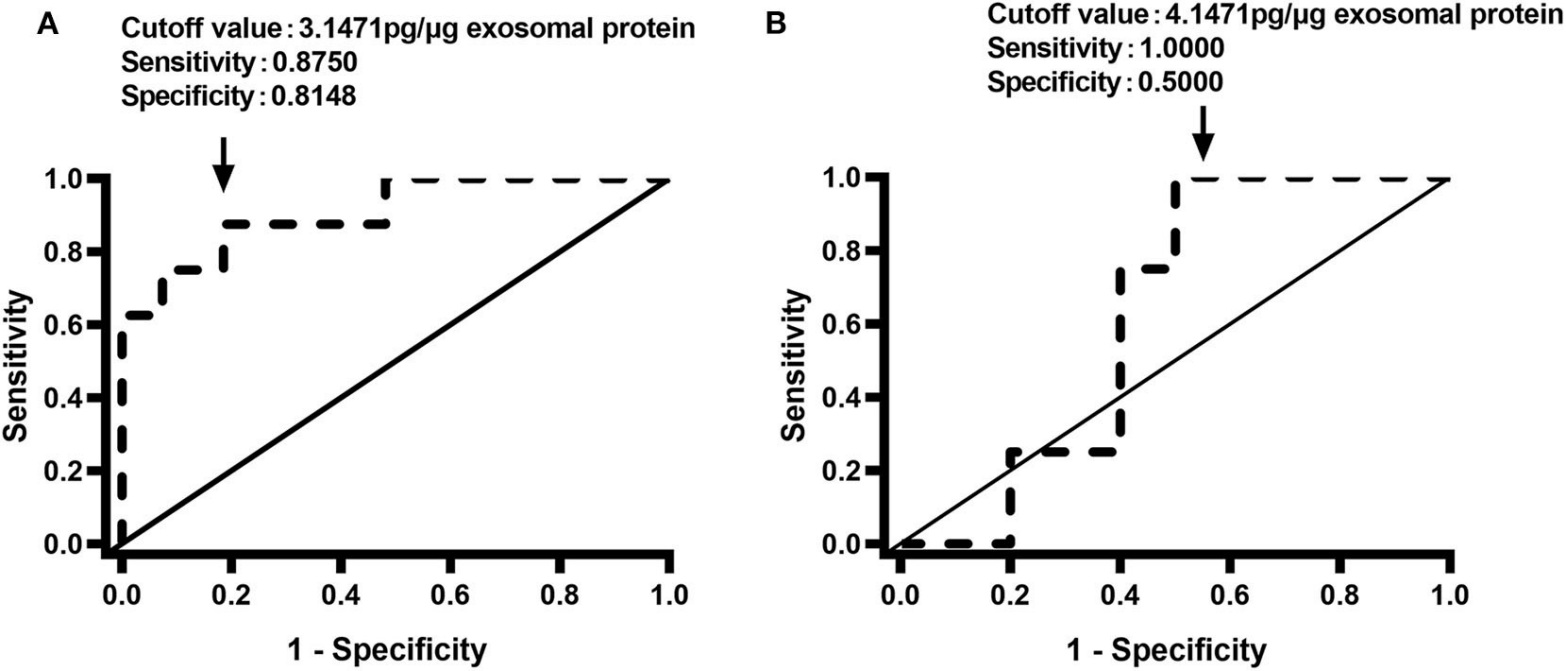
Receiver operating characteristic curves for the discrimination of DRF <30% using the urinary exosome level of MXRA5. **(A)** Exosomal level of MXRA5 in BU (area under the curve = 0.9074). **(B)** Exosomal level of MXRA5 in KU (area under the curve = 0.625).

We also determined the relationships between the renal pelvis and bladder MXRA5 levels and DRF. There were no significant correlations between the whole-urine MXRA5 levels in either the BU or KU samples and DRF (*R*^2^ = 0.1115, *P* = 0.05, and *R*^2^ = 0.3313, *P* = 0.0502, respectively) (Figures 4A,B). In contrast, the exosomal MXRA5 levels in the BU samples significantly correlated with DRF (*R*^2^ = 0.69, *P* < 0.0001), and the strongest correlation was between the exosomal MXRA5 levels in the KU samples and DRF (*R*^2^ = 0.8128, *P* < 0.0001) (Figures 4C,D). Figures 4E,F show the results of univariate binary logistic regression analyses, which demonstrated that the exosomal MXRA5 level was not associated with the APD. ROC curve analysis showed that the cut-off values for the exosomal MXRA5 level in BU and the exosomal MXRA5 level in KU were 3.1471 pg/μg exosomal protein (sensitivity 87.5%, specificity 81.5%) and 4.1471 pg/μg exosomal protein (sensitivity 100%, specificity 50%) with a discrimination of DRF<30%, respectively, and the area under curve (AUC) was 0.9074 (*P* = 0.0005) and 0.6250 (*P* = 0.4795), respectively (Figures 5A,B).

## Discussion

Children with UPJO often experience long-term sequelae before any surgical intervention and are difficult for pediatric surgeons to evaluate (22, 23). Methods that are capable of monitoring renal damage in UPJO patients would substantially aid the diagnosis of patients at risk of a deterioration in renal function and permit the monitoring of treatment efficacy. Although routine laboratory tests, invasive radiographic imaging, and repeated ultrasonography are widely used to evaluate UPJO-induced kidney damage (24), the measurement of urine biomarkers of renal damage now has a wide range of clinical applications.

The recent identification of urinary biomarkers provides promising diagnostic and prognostic tools for renal injury. Furthermore, urinary biomarkers could theoretically help to identify a loss of kidney function in patients with unilateral UPJO (25, 26). Several potential candidate urine biomarkers have been identified in the past decades. Nicholas et al. described the use of urinary neutrophil gelatinase-associated lipocalin (NGAL) as a sensitive biomarker of renal injury in the context of UPJO (15). They showed that urinary NGAL levels correlate with DRF in the affected kidney in patients with unilateral UPJO, and could thus be used as a sensitive biomarker of kidney injury in children with UPJO. In addition, Katarzyna et al. demonstrated higher renal activities of exoglycosidases, which are indicative of renal tubular damage, in children with UPJO (12). Finally, increasing urinary procollagen III N-terminal propeptide protein levels were also shown to be associated with worsening obstruction in children (27). Thus, the measurement of urine proteins as indices of renal injury has a wide range of potential clinical applications. Clinical interest has focused on the potential uses of urine markers that are associated with various aspects of the pathophysiology of UPJO, such as inflammation, apoptosis, oxidative stress, and fibrosis (10, 13, 15, 27). UPJO is characterized by tubulointerstitial fibrosis in the obstructed kidney, and as previously demonstrated, high levels of inflammation- and fibrosis-related biomarkers in the urine could suggest renal impairment (27–29).

MXRA5 is a 312-kDa adhesion protein that is expressed abundantly in human renal tissue. MXRA5 has also been described to be a promising marker of early tissue injury and fibrosis (19), and its application has been extensively investigated in experimental studies of myocardial damage, kidney cancer, and chronic kidney disease (19, 30). Urine MXRA5 concentrations may provide a sensitive marker of early kidney damage in children with UPJO-induced hydronephrosis and there may be a relationship between MXRA5 concentration and disease severity. We evaluated this possibility in the present study by measuring the MXRA5 concentrations in urine samples from 35 infants with unilateral UPJO and 12 healthy controls.

We initially analyzed whole-urine MXRA5 levels and found no significant difference between controls and UJPO patients in either their bladder or renal pelvic samples. This may be because of the relatively small number of participants, and means that we cannot draw an unequivocal conclusion, meaning that further validation is required in a larger study. Exosomes, which are generated by cells in various nephron segments and parts of the urinary tract, are abundant in the urine. Urinary exosomes, as a potential reservoir of urine biomarkers, may reflect the underlying changes in the kidney tissue better than whole-urine samples. This raises the possibility that urinary exosomal proteins may prove to be more sensitive biomarkers that whole-urine proteins.

Following this rationale, we analyzed the MRXA5 levels in urinary exosomes and showed that they were significantly higher in patients with UPJO than in normal controls. Whole-urine protein measurements may be confounded by the presence of plasma proteins, whereas urinary exosomes may be more representative of the urinary tract (31, 32), and may thus provide more valuable insights into renal injury status than the analysis of whole urine. Our results indicate that MXRA5 in urine exosomes has potential utility in the diagnosis of UPJO-induced kidney injury.

DRF is the most commonly used measure of the severity of renal injury in UPJO (33). Therefore, we assessed the relationship between urinary MXRA5 level and DRF. We found no significant correlation between whole-urine MXRA5 level and DRF; however, the urinary exosomal MXRA5 level could be used to predict renal function in patients with UPJO. These results imply that proteins in urinary exosomes may reflect the mechanism of renal injury more accurately than similar proteins in whole-urine samples. ROC curve analysis showed that MXRA5 level in BU had a great predictive marker for children with UPJO with DRF<30%, but MXRA5 level in KU didn't because of limited cases probably. These findings corroborate the usefulness of urinary exosomal MXRA5 as a non-invasive marker of lower ipsilateral DRF in UPJO patients. However, it may not reflect the severity of obstruction because of the lack of patients who were undergoing conservative treatment and had mild hydronephrosis in the present study. Moreover, MXRA5 may not reflect the state of kidney function during this period of intense growth and metabolic activity. All these factors limit the clinical usefulness of the measurement of exosomal MXRA5 level in patients with UPJO at the present time. Further studies are required that include a sufficient number of controls to accurately identify the appropriate urinary exosomal MXRA5 cut-off levels.

The genesis and sources of urinary exosomal MXRA5 in patients with UPJO-induced kidney injury require further clarification. The upregulation of TGF-β1 is the key factor responsible for the pathophysiological changes in human obstructive renal injury (29). TGF-β1 has been shown to upregulate MXRA5 expression in proximal tubular cells (19). In contrast, MXRA5 also behaves as an anti-inflammatory and anti-fibrotic molecule in tubular cells (19). The human kidney has relatively high levels of MXRA5 compared with other organs, and the injured kidney may thus be a major source of MXRA5. The high expression of MXRA5 in urine exosomes in the present study suggests that it plays a biological role in the pathogenesis of UPJO.

Urine is a reliable source of renal injury markers and many candidate markers of UPJO-induced renal injury have been identified. Researchers maintain that the analysis of biomarkers in the urine is highly desirable because it can be collected non-invasively and easily (10–12, 15, 34). The present results show that MXRA5 in urinary exosomes is more strongly correlated with UPJO than whole-urine MXRA5, which implies it may be useful for the identification of UPJO-induced kidney injury. However, exosome isolation is time consuming, expensive, impractical for everyday clinical use, and may be limited by low yields. Moreover, exosome evaluation is also much more complicated (35, 36). Therefore, a more rapid and efficient method of extracting exosomes from urine samples should be sought urgently.

The present study had several limitations. First, it was limited to one hospital and the sample size was small. Future studies should be multi-centric and include a larger number of patients with UPJO and controls. However, by comparing the patients with an age and sex-matched control group, we believe that we have provided robust evidence that MRXA5 is a non-invasive biomarker of renal damage. Second, MXRA5 levels were not measured after hydronephrosis had improved, and it would be helpful to assess the relationship between MXRA5 level and clinical end-points during follow up. Third, MXRA5 should also be evaluated longitudinally in the future, to better understand its role in normal renal development. Finally, we did not compare the urinary MXRA5 levels among patients with different glomerular filtration rates and were therefore unable to assess its relationship with bladder MXRA5. However, despite these limitations, the results of the present study imply that urinary exosomal MXRA5 may represent a biomarker of UPJO.

## Conclusion

The present findings strongly suggest that urinary MXRA5 is a potential biomarker of renal injury. In addition, higher levels of urinary exosomal MXRA5 significantly correlated with lower DRF. The use of a combination of clinical characteristics and the non-invasive analysis of urinary MXRA5 may help doctors make an accurate diagnosis and predict the clinical evolution of UPJO. However, further studies are required to clarify the changes in the kidney that associate with alterations in the exosomal proteome. Overall, urinary exosomal analysis appears to provide an attractive approach to the diagnosis and monitoring of UPJO-induced renal injury. However, there are still several limitations to be circumvented before urinary exosomal markers become clinically established.

## Biosecurity Statement

All the standard biosecurity and institutional safety procedures were adhered to during the experimental procedures described in this article.

## Data Availability Statement

All datasets presented in this study are included in the article/supplementary material.

## Ethics Statement

The studies involving human participants were reviewed and approved by the Shanghai Children's Medical Center Research Ethics Committee. Written informed consent to participate in this study was provided by the participants' legal guardian/next of kin.

## Author Contributions

DJ, QW, and ZS contributed to conception and design of the study. QW, XX, and ZS participated in the performance of the research. YD, WL, and TX performed the statistical analysis. DJ wrote the first draft of the manuscript. QW and ZS wrote sections of the manuscript. All authors contributed to manuscript revision, and read, and approved the submitted version.

## Conflict of Interest

The authors declare that the research was conducted in the absence of any commercial or financial relationships that could be construed as a potential conflict of interest.
